# Ten rapid antigen tests for SARS-CoV-2 widely differ in their ability to detect Omicron-BA.4 and -BA.5

**DOI:** 10.1007/s00430-023-00775-8

**Published:** 2023-08-10

**Authors:** Franziska Krenn, Christopher Dächert, Irina Badell, Gaia Lupoli, Gamze Naz Öztan, Tianle Feng, Nikolas Schneider, Melanie Huber, Hanna Both, Patricia M. Späth, Maximilian Muenchhoff, Alexander Graf, Stefan Krebs, Helmut Blum, Jürgen Durner, Ludwig Czibere, Lars Kaderali, Oliver T. Keppler, Hanna-Mari Baldauf, Andreas Osterman

**Affiliations:** 1 Max von Pettenkofer Institute & Gene Center, Virology, National Reference Center for Retroviruses, LMU München, Munich, Germany; 2 German Center for Infection Research (DZIF), Partner Site, Munich, Germany; 3 COVID-19 Registry of the LMU Munich (CORKUM), University Hospital, LMU München, Munich, Germany; 4 Laboratory for Functional Genome Analysis, Gene Center, LMU München, Munich, Germany; 5 Labor Becker MVZ GbR, Munich, Germany; 6 Institute of Bioinformatics, University Medicine Greifswald, Greifswald, Germany

**Keywords:** SARS-CoV-2 rapid antigen test, Nucleocapsid protein, Diagnostic test, Sensitivity, Specificity, VoC, Lateral flow, Omicron

## Abstract

Since late 2021, the variant landscape of the severe acute respiratory syndrome coronavirus 2 (SARS-CoV-2) has been dominated by the variant of concern (VoC) Omicron and its sublineages. We and others have shown that the detection of Omicron-BA.1 and -BA.2-positive respiratory specimens by rapid antigen tests (RATs) is impaired compared to Delta VoC-containing samples. Here, in a single-center retrospective laboratory study, we evaluated the performance of ten most commonly used RATs for the detection of Omicron-BA.4 and -BA.5 infections. We used 171 respiratory swab specimens from SARS-CoV-2 RNA-positive patients, of which 71 were classified as BA.4 and 100 as BA.5. All swabs were collected between July and September 2022. 50 SARS-CoV-2 PCR-negative samples from healthy individuals, collected in October 2022, showed high specificity in 9 out of 10 RATs. When assessing analytical sensitivity using clinical specimens, the 50% limit of detection (LoD50) ranged from 7.6 × 10^4^ to 3.3 × 10^6^ RNA copies subjected to the RATs for BA.4 compared to 6.8 × 10^4^ to 3.0 × 10^6^ for BA.5. Overall, intra-assay differences for the detection of these two Omicron subvariants were not significant for both respiratory swabs and tissue culture-expanded virus isolates. In contrast, marked heterogeneity was observed among the ten RATs: to be positive in these point-of-care tests, up to 443-fold (BA.4) and up to 56-fold (BA.5) higher viral loads were required for the worst performing RAT compared to the best performing RAT. True-positive rates for Omicron-BA.4- or -BA.5-containing specimens in the highest viral load category (*C*_t_ values < 25) ranged from 94.3 to 34.3%, dropping to 25.6 to 0% for samples with intermediate *C*_t_ values (25–30). We conclude that the high heterogeneity in the performance of commonly used RATs remains a challenge for the general public to obtain reliable results in the evolving Omicron subvariant-driven pandemic.

**Supplementary Information:**

The online version contains supplementary material available at 10.1007/s00430-023-00775-8.

## Introduction

Since the start of the SARS-CoV-2 pandemic in early 2020, several VoCs have emerged, which differ in their biological characteristics such as transmissibility or virulence from the original Wuhan strain [1]. The Omicron VoC has now been circulating since the end of 2021 with different sublineages evolving over time. Globally, Omicron subvariants BA.5, XBB.1.5, BF.7, BQ.1., and BQ.1.1 are currently responsible for the majority of SARS-CoV-2 infections [2, 3].

To diagnose SARS-CoV-2 infections, rapid antigen tests (RATs) were implemented next to the gold-standard nucleic acid amplification tests (NAATs). At the beginning of the pandemic, NAATs, such as quantitative reverse transcription polymerase chain reactions (qRT-PCRs), quickly reached their capacity limits due to supply shortage [4]. However, the performance of individual RATs may in part depend on their ability to detect mutations in the nucleocapsid protein of emerging VoCs [5]. Moreover, increasing vaccine- and infection-mediated immunity or clinical characteristics of novel VoCs or sublineages may affect the performance of RATs. Of note, criteria for effective health care interventions such as RATs set by the World Health Organization (WHO), especially those for layman ‘s use, encompass a minimal sensitivity of 80% and minimal specificity of 97% [6]. The council of the European Union has recently even increased the minimal sensitivity to 90% [7]. It is thus of utmost importance that RATs are constantly re-evaluated by independent laboratories during the evolving pandemic.

The performance of various RATs has been evaluated by us and others revealing a high variability for detection of SARS-CoV-2-positive swabs and, in particular, the failure of a number of tests to detected certain VoCs [8–43]. In 2022, the Paul Ehrlich Institute in Germany re-assessed the efficacy of RATs for layman’s use using a small panel of pooled respiratory specimen containing Omicron-BA.1 as well as a predictive epitope-based “bridging evaluation” [44]. These findings were also included in an updated “common list” of RATs published by the European Union [45]. It has to be taken into account though that this information does not necessarily translate into reliable information on the performance of these tests for currently circulating Omicron sublineages. Differences in the performance of RATs in recognizing individuals infected with Omicron sublineages, which have emerged and rapidly diversified in 2022, have thus far only been addressed by a small number of studies [14, 30, 33, 42, 46–48], and not yet for Omicron-BA.4 and -BA.5. The aim of the current study was to compare the performance of ten commercially available RATs to detect respiratory samples from COVID-19 patients infected with either BA.4 or BA.5**.**

## Materials and methods

### Respiratory swabs

The sample collection was performed in analogy to our previous studies [42] where detailed descriptions can be found. In brief, the study center of the Max von Pettenkofer Institute was provided with anonymized SARS-CoV-2 PCR-positive swab samples from Becker MVZ GbR laboratory, a regional diagnostic laboratory in which the respiratory swabs were also characterized as BA.4- or BA.5-positive samples by variant-specific PCR (see below). Respiratory swabs were obtained from various medical facilities (hospitals, family practices, nursing homes, etc.). No further information, such as sampling site or disease status, was available. After receiving the swabs, qRT-PCR was conducted within 24 h to determine the viral load (see below). Subsequently, depending on the detected viral load, a random selection of suitable samples was performed as recommended by the Technical working group on COVID-19 diagnostic tests of the European Commission (EC): The composition of the reference panel covers SARS-CoV-2 viral loads ranging from approximately 4.2 × 10^2^ to 1.1 × 10^9^ genome copies per mL of specimens and *C*_t_ values between 36 and 17, respectively. These frame values were applied to a viral load-based conversion of the Ct values obtained in our laboratory (Suppl. Table 1). Following recommendations by the PEI and European Commission [49, 50], three subgroups were defined within Ct value ranges of "very high" (viral load 1.1 × 10^9^–2.1 × 10^6^), "high" (viral load 2.1 × 10^6^–4.4 × 10^4^), and "medium" (viral load 4.4 × 10^4^–4.1 × 10^2^) [51].

A total of 171 SARS-CoV-2 PCR-positive respiratory swabs (Omicron-BA.4: 71, Omicron-BA.5: 100) were examined between 07/27/2022 and 09/23/2022 in a retrospective in vitro study. Following EC recommendations, Omicron-BA.5-positive swabs were selected such that 35% fell into viral load category "very high,” 45% into category "high," and 20% into category "medium." Of note, since the study period coincided with the transition from Omicron-BA.4 dominating to -BA.5 in Bavaria, there were not sufficient numbers of BA.4-positive swab samples available to achieve a comparable representation. Respiratory samples were stored at 2–8 °C prior to testing for up to 48 h.

### SARS-CoV-2 rapid antigen tests

In order to evaluate the sensitivity of the respective assays, a defined volume (50 µl) of the virus-containing sample material (liquid transport medium) was transferred to the RATs by trained staff. This volume was completely absorbed by the swabs enclosed within each RAT. Based on this established protocol, the presented results refer to "RNA copies subjected to the test." According to the manufacturer information, the inoculated swabs were transferred to the lysis buffers and the specified amount of material was added to the appropriate test cassette. This procedure is in accordance with an internationally accepted method for transferring virus transport medium (VTM) applied to the inoculation swab solution into the test cassette [50]. After 15 min, the reading of the results was performed with blinded information about the viral load present in the sample under constant light conditions. Any visible test line—regardless of its intensity—was considered positive. Invalid tests were excluded.

The following 10 tests were examined in our study: Wantai SARS-CoV-2 Ag Rapid Test (colloidal gold) (Beijing WANTAI Biological Pharmacy Enterprise Co.) (“Wantai”), Clinitest Rapid COVID-19 Antigen Test (Siemens Healthineers/Healgen Scientific Limited Liability Company) (“Siemens”), Coronavirus (2019-nCoV) Antigen Test (Beijing Hotgen Biotech Co.) (“Hotgen”), SARS-CoV-2 Antigen Test Kit (Colloidal Gold) (Genrui Biotech Inc.) (“Genrui”), COVID-19 Antigen Rapid Test (Colloidal Gold) Cassette (Joinstar Biomedical Technology Co.) (“Joinstar”), COVID-19 Antigen Detection Kit (New Gene (Hangzhou) Bioengineering Co.) (“New Gene”), COVID-19 Antigen Rapid test Kit (Colloidal Gold) (Xiamen AmonMed Biotechnology Co.) (“AmonMed”), COVID-19 Antigen Rapid Test Kit (Hangzhou Clongene Biotech Co., Ltd.) (“Clongene”), iHealth COVID-19 Antigen Rapid Test (iHealth Labs, Inc.) (“iHealth”), and NADAL COVID-19 Ag Rapid Test (nal von Minden GmbH) (“nal von Minden”).

Except for iHealth, all tests are included in the EU Common list of COVID-19 antigen tests. Wantai and Siemens correspond to category A.1 (eligible COVID-19 RATs for which their performance has been evaluated through prospective clinical field studies), the others to category B.1 (eligible COVID-19 RATs for which their performance has been evaluated through retrospective in vitro studies) according to the EU common list of COVID-19 antigen tests [45]. At the time of this study, nal von Minden had been evaluated by the Paul Ehrlich Institute (PEI) but was not yet included in the EU Common list. It is now included in the category A.1. iHealth is among the FDA-authorized over-the-counter tests [52].

Due to frequent changes in national and international testing strategies, political decisions on the use, and reimbursement of RATs or special sales in discounter retail chains, the popularity of individual RATs varied considerably. Therefore, we based the test selection for the current study on the following evidence to investigate tests relevant for the general population: two tests with high sensitivities were selected from the previous study [42]—Clongene and nal von Minden. Based on data provided by the Quarterly analysis 01/22 by APO Fusion Plus 2021 (INSIGHT Health GmbH & Co. KG) as well as sales statistics of two leading pharmaceutical major distributors (pharmaceutical wholesalers Sanacorp pharmaceutical trade GmbH (Sanacorp Pharmahandel GmbH) and Noweda pharmacy cooperative eG (Noweda Apothekergenossenschaft eG)), we included Hotgen, Joinstar, AmonMed, Wantai, and Genrui. Ranked as bestsellers at the time of study initiation by the online retailer amazon.com (bestsellers from amazon.de are already included above) were iHealth and Siemens. According to the Austrian Ministry of Health, a free RAT available in Austrian pharmacies for self-application ("living room tests") was New Gene [53].

### PCR screening and SARS-CoV-2 variant-specific PCR

Using the method of the previously published “Munich Extraction Protocol” [54], SARS-CoV-2 PCR-positive samples were identified by the Becker MVZ BgR laboratory. A variant-specific PCR (modified version of the COVID-19 direct RT-PCR kit (FRIZ Biochem GmbH, Neuried, Germany)) was used to determine the SARS-CoV-2 variant. Samples corresponding to the Omicron-BA.4 and -BA.5 sublineages were sent to the Max von Pettenkofer Institute for further analyses. In order to keep the time between sample collection and test performance as short as possible, variant-specific PCR was the method of choice as the results correlate well with those from whole genome sequencing [55]. At the time of the study, epidemiological surveillance showed that > 60% of the Omicron-BA.5-positive samples detected belonged to the Omicron-BA.5.1 and -BA.5.2 sublineages [56].

### Quantitative viral load determination

For the quantification of viral load, the Roche Cobas SARS-CoV-2 E gene reaction of a Cobas 6800 system (Roche Diagnostics GmbH, Mannheim, Deutschland) of the accredited routine diagnostics laboratory of the Max von Pettenkofer Institute was exclusively used. A detailed description of the formula used for the calculation can be found in previous studies [42]. Variations in values between different qRT-PCR runs were not taken into account. However, as this affects all results, it does not influence their interpretation.

### Expansion of SARS-CoV-2 from primary patient material

Omicron isolates from primary patient material were propagated on Vero-E6 cells (CRL-1586, (American Type Culture Collection, ATCC, Virginia, USA). Otherwise, the protocol of cell culture derived samples is consistent with the previously described method [33]. Expanded stocks of Omicron-BA.4 and -BA.5 were classified by next generation sequencing (NGS) (B.1.1.529.4: GISAID EPI ISL: hCoV-19/Germany/BY-MVP-000015293/2022; B.1.1.529.5: GISAID EPI ISL: hCoV-19/Germany/BY-MVP-000015294/2022) and RNA copies per mL were determined as the mean from three independent biological experiments with technical unicates or duplicates. For Omicron-BA.4, two defining mutations of the nucleocapsid protein N:P151S and N:S413R were detected by NGS analysis; for Omicron-BA.5, the N:S413R mutation was detected. Neither the Omicron-BA.4 nor -BA.5 isolate carried the N:E136D mutation.

### RAT specificity

To determine RAT’s specificity, healthy volunteers performed naso- or oropharyngeal PCR swabs (Copan eSwabTM, COPAN ITALIA Brescia, Italy). From these individual samples, pooled samples were created: Depending on the number of samples in the pool, 100 µl (for pools of 4 to max. 8 subjects) or 200 µl (for pools of 2–3 subjects) of the VTM was combined to form a respective pool sample. These combined samples were analyzed for the presence of SARS-CoV-2 RNA using the Xpert Xpress SARS-CoV-2 plus GeneXpert system (Cepheid Inc.; Sunnyvale, California, USA). Individual samples from participants of pool samples with positive results were retested as individual samples; participants were subsequently informed about the SARS-CoV-2-positive result, reported to the responsible local health authorities according to the current laws, and excluded from this study. Volunteers with negative individual samples as well as negative pool samples subsequently performed 5–10 RATs within 1–6 h after PCR swab collection and results were anonymized. RATs were performed according to the manufacturers’ instructions under the supervision of trained personnel. Reading and interpretation of the results were performed according to criteria described above.

### Statistical analyses

Statistical analyses were performed in R version 4.1.2. Binomial confidence intervals for sensitivities and specificities were computed using the Wilson score interval. To further analyze analytical sensitivities, we used logistic regression, with viral loads and RNA copy numbers subjected to the test as independent and test outcomes as the dependent variable, yielding detection probabilities for each viral load level.

## Results

### Evaluation of RAT specificity

Extending our previous work for Omicron-BA.1 and -BA.2 [33, 42], the current study evaluated the performance of ten RATs for detecting Omicron-BA.4 and -BA.5 in respiratory swabs. We chose RATs based either on their performance in our previous study [42], bestseller lists of pharmacies, pharmaceutical wholesalers or an online retailer. Except for iHealth, all RATs were freely available on the European market. First, we determined the specificity of the ten RATs using respiratory swabs of healthy, SARS-CoV-2 PCR-negative volunteers (Table 1). The specificity ranged from 84 to 100%, with AmonMed being the only RAT not fulfilling WHO’s specificity criteria with > 97% [6].

**Table 1:** Determination of assay specificity for ten qualitative SARS-CoV-2 rapid antigen tests using SARS-CoV-2 PCR-negative respiratory swabs from adults

Assay	Specificity (%)	95% CI	True negative/total
iHealth	100.00	92.87–100.00	50/50
Clongene	100.00	92.87–100.00	50/50
nal von Minden	100.00	92.87–100.00	50/50
Hotgene	100.00	92.87–100.00	50/50
Joinstar	100.00	92.87–100.00	50/50
Siemens	100.00	92.87–100.00	50/50
New Gene	100.00	92.87–100.00	50/50
Wantai	100.00	92.87–100.00	50/50
Genrui	97.92	89.10–99.89	47/48
AmonMed	84.00	71.49–91.66	42/50

### Analytical sensitivity of RATs for detecting Omicron-BA.4 and -BA.5 in clinical specimens

**Fig. 1:**
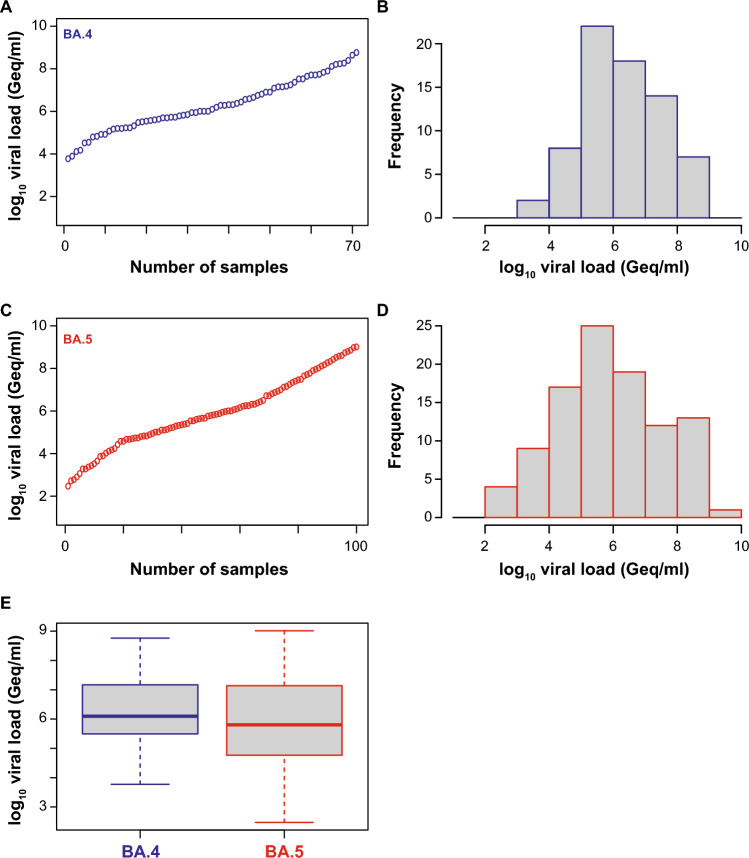
SARS-CoV-2 viral load distribution of respiratory samples included in this study. **A**, **C** Shown is the log10 viral load (Geq/ml) of 71 SARS-CoV-2-positive Omicron-BA.4 (**A**, blue) and 100 SARS-CoV-2-positive Omicron-BA.5 (**C**, red) patient samples, sorted by ascending magnitude of the viral load from left to right. Each dot indicates one patient and the sample’s ID is indicated. **B, D** Depicted is the histogram of the viral load distribution for Omicron-BA.4 (**B**, blue) and for Omicron-BA.5 (**D**, red) by categorization of samples into defined log10 viral load ranges. Each histogram bar indicates the number of samples in the respective viral load range. **E** Depicted is the comparison of the samples shown in **A** and **C**. The horizontal line in the box plots shows the median of the samples, bound between upper and lower quartiles, and whiskers between minimum and maximum are indicated

Next, we used 171 nasal/nasopharyngeal swabs from SARS-CoV-2 RNA-positive patients, of which 71 were classified by variant-specific PCR and whole genome sequencing as BA.4 and 100 as BA.5, in principle as reported [57], to evaluate the analytical sensitivity of these RATs. Viral loads ranged between 5,949 and 582,434,112 Geq/ml for BA.4 (median: 1,257,020 Geq/ml) and 297 and 1,030,950,803 Geq/ml for BA.5 (median: 638,453 Geq/ml), respectively (Fig. 1).

We then determined the individual RAT’s sensitivity by scoring the frequency of true-positive respiratory samples. The overall assay sensitivities ranged between 17 and 49% (Tables 2 and 3). Among the ten RATs evaluated, iHealth performed best: This test was slightly superior in detecting BA.4-positive samples (49%) compared to BA.5-positive swabs (44%). nal von Minden detected 39% of all BA.4 samples and 36% of all BA.5 samples. For AmonMed, the sensitivity for BA.5 (43%) was slightly higher than for BA.4 (37%). A similar trend was found for Clongene (34% BA.4 vs. 40% BA.5). New Gene had similar sensitivities for both Omicron subvariants with around 36%. The analytical sensitivities for Siemens (BA.4 (31%), BA.5 (30%)), Joinstar (BA.4 (30%), BA.5 (32%)), and Hotgene (BA.4 (27%), BA.5 (30%)) were comparable. Genrui performed poorly with sensitivities of 21% (BA.4) and 22% (BA.5), respectively. Wantai performed worst among the ten RATs examined scoring only 17% (BA.4) and 18% (BA.5) of PCR-positive samples truly positive.

**Table 2:** Determination of assay sensitivity for ten SARS-CoV-2 rapid antigen tests in SARS-CoV-2 PCR-positive respiratory swabs classified as Omicron-BA.4

Assay	Sensitivity (%)	95% CI	True positive/total
iHealth	49.30	38.01–60.66	35/71
nal von Minden	39.44	28.89–51.06	28/71
AmonMed	36.62	26.37–48.24	26/71
New Gene	36.62	26.37–48.24	26/71
Clongene	33.80	23.88–45.38	24/71
Siemens	30.99	21.44–42.48	22/71
Joinstar	29.58	20.23–41.02	21/71
Hotgene	26.76	17.84–38.05	19/71
Genrui	21.13	13.24–31.97	15/71
Wantai	16.90	9.94–27.26	12/71

**Table 3:** Determination of assay sensitivity for ten SARS-CoV-2 rapid antigen tests in SARS-CoV-2 PCR-positive respiratory swabs classified as Omicron-BA.5

Assay	Sensitivity (%)	95% CI	True positive/total
iHealth	44.00	34.67–53.77	44/100
AmonMed	43.00	33.73–52.78	43/100
Clongene	40.00	30.94–49.80	40/100
nal von Minden	36.00	27.27–45.76	36/100
New Gene	36.00	27.27–45.76	36/100
Joinstar	32.00	23.67–41.66	32/100
Hotgene	30.30	22.13–39.95	30/99
Siemens	30.00	21.89–39.58	30/100
Genrui	22.00	15.00–31.07	22/100
Wantai	18.00	11.70–26.67	18/100

**Fig. 2:**
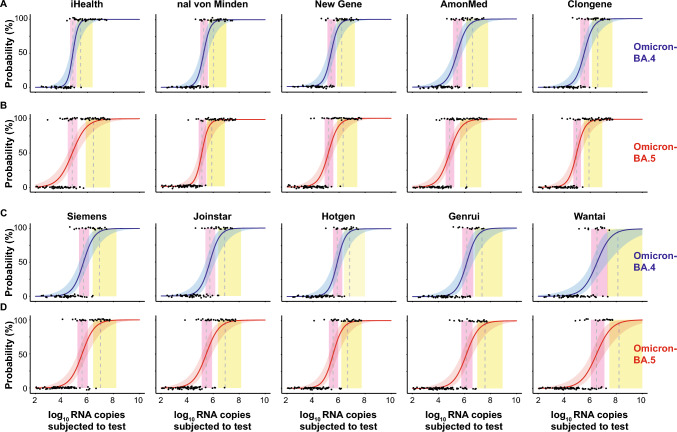
Limit of detection analyses of RT-qPCR-positive respiratory samples positive for either Omicron-BA.4 (**A, C**, blue) or Omicron-BA.5 (**B**, **D**, red) using ten SARS-CoV-2 RATs. The log10 RNA copies subjected to the test of quantified samples on the x axis were plotted against a positive (+ 1) or negative (0) test outcome on the y axis. For readability of the figure, slight normal jitter was added to the y values. Blue/red curves show logistic regressions of the viral load on the test outcome; vertical dashed lines indicate log viral loads at which 50% (LoD50) and 95% (LoD95), respectively, of the samples are expected positive based on the regression results

In line with previous reports [9, 33, 42], we calculated the 50% (dotted line in pink vertical area) and 95% (dotted line in yellow vertical area) limit of detection (LoD) using a logistic regression model (Fig. 2A,C: BA.4; Fig. 2B,D: BA.5). Similar to BA.1 and BA.2 [42], no statistically significant differences for the detection of BA.4- and BA.5-containing respiratory samples were noted for individual RATs despite slight differences in LoD50/LoD95 values. iHealth had LoD50 and LoD95 values corresponding to 75,500 and 297,107 RNA copies for BA.4 (Fig. 2A), respectively, and 67,995 (LoD50) and 2,876,315 (LoD95) RNA copies for BA.5 (Fig. 2B), respectively. Remarkably, LoD95 values for iHealth differed between BA.4 and BA.5 by about tenfold. nal von Minden was quite comparable to iHealth in terms of LoD50/LoD95 values for both BA.4 and BA.5. The LoD50 and LoD95 values equaled 184,181 and 1,063,640 SARS-CoV-2 RNA copies for BA.4 (Fig. 2A) and 135,578 (LoD50) and 778,616 (LoD95) RNA copies for BA.5 (Fig. 2B), respectively. In contrast, New Gene was inferior to iHealth and showed up to fivefold higher LoD values. The LoD50 and LoD95 values for New Gene were 245,795 and 1,603,684 RNA copies for BA.4, respectively (Fig. 2A). The LoD50 and LoD95 values for BA.5 equaled 154,671 and 2,070,678 RNA copies (Fig. 2B), respectively, and were in a similar range compared to BA.4. AmonMed had an up to fourfold difference in detecting BA.4 and BA.5. The LoD50 and LoD95 values for BA.4 using AmonMed corresponded to 271,387 and 4,057,302 RNA copies (Fig. 2A), respectively, whereas they were 71,011 (LoD50) and 1,431,929 (LoD95) RNA copies for BA.5 (Fig. 2B). The LoD50 and LoD95 values for Clongene were comparable to AmonMed with 346,916 and 3,778,936 RNA copies for BA.4 (Fig. 2A), respectively, and for BA.5 90,362 (LoD50) and 801,777 (LoD95) RNA copies (Fig. 2B) Interestingly, Clongene was inferior to iHealth for BA.4 detection (up to 13-fold), but similar or even slightly better than iHealth to score BA.5 samples as positive. This was also reflected by the 4- to fivefold difference in LoD50 and LoD95 values for Clongene between BA.4 and BA.5 samples, despite not reaching statistical significance. Values obtained for Siemens and Joinstar were quite similar for both BA.4 and BA.5. For Siemens, the LoD50 and LoD95 values for BA.4 were 493,158 and 8,386,261 RNA copies (Fig. 2C) and for BA.5 385,879 (LoD50) and 9,980,728 (LoD95) RNA copies (Fig. 2D), respectively. Joinstar had LoD50 and LoD95 values for BA.4 of 558,024 and 7,627,405 RNA copies (Fig. 2C), and for BA.5 294,868 (LoD50) and 8,384,834 (LoD95) RNA copies (Fig. 2D), respectively. Compared to iHealth, Hotgene was up to 22-fold inferior to detect BA.4, but only up to fivefold inferior for detecting BA.5-positive samples. The LoD50 and LoD95 values for BA.4 corresponded to 725,768 and 6,419,779 RNA copies (Fig. 2C) and for BA.5 347,660 and 4,465,459 RNA copies, respectively (Fig. 2D). Among the ten RATs, Genrui performed second worst. The LoD50 and LoD95 values for BA.4 were up to 74-fold and for BA.5 up to 20-fold higher compared to iHealth. For BA.4, the LoD50 and LoD95 values corresponded to 1,497,510 and 21,927,942 RNA copies (Fig. 2C), respectively. For BA.5, those values equaled 1,356,891 (LoD50) and 37,276,411 (LoD95) RNA copies (Fig. 2D), respectively. Looking at the LoD analyses, Wantai performed worst. For this RAT, the LoD50 and LoD95 values were up to 443-fold higher for BA.4 and up to 56-fold for BA.5 when compared to iHealth. The LoD50 and LoD95 for BA.4 equaled 3,267,974 and 131,653,605 RNA copies (Fig. 2C), respectively, and for BA.5 3,049,324 (LoD50) and 162,296,370 (LoD95) RNA copies (Fig. 2D).

### Analytical RAT sensitivity in cell culture-expanded virus stocks

Similar to our previous reports [33], we used cell culture-expanded virus stocks of Omicron-BA.4 and -BA.5. Whole genome sequencing confirmed the sublineage-specific point mutations N:P151S and N:S413R for BA.4 and N:S413R for BA.5, respectively. None of the expanded patient isolates showed the N:E136D mutation.

**Fig. 3:**
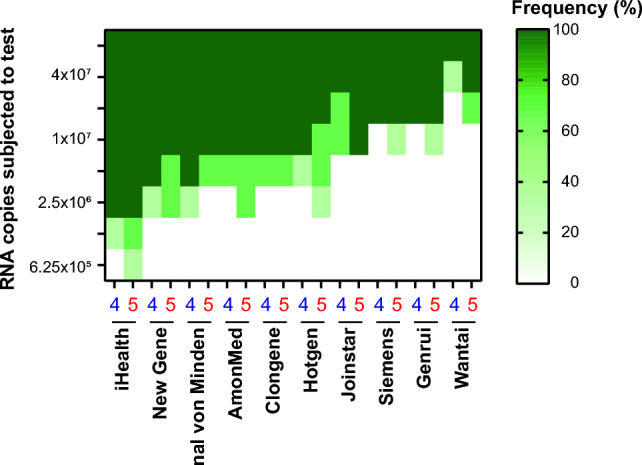
Heat map of analytical sensitivity of ten SARS-CoV-2 RATs for SARS-CoV-2 variants Omicron-BA.4 and -BA.5 expanded in cell culture. The visual read of triplicate samples were plotted against the calculated viral RNA copies subjected to the test. Dark green: Positive RAT result; Light green: Replicate mixed result; White: Negative RAT result. 4: Omicron-BA.4; 5: Omicron-BA.5

The variability among the ten RATs to score these cell culture-expanded isolates positive was high (Fig. 3). We noted a slight overall trend toward a better detection of BA.5 over BA.4. Mirroring the results obtained for detection of PCR-positive swabs, Wantai performed worst also in recognizing tissue culture-expanded virus isolates. This RAT was only able to score positive using an input equivalent of 4 × 10^7^ RNA copies for BA.4 and 2 × 10^7^ RNA copies for BA.5. The majority of RATs was able to detect both BA.4 and BA.5 between 2.5 × 10^6^ and 1 × 10^7^ RNA copies. iHealth performed best requiring 16-fold (BA.5) to 32-fold (BA.4) lower viral RNA concentrations in the input virus stock to score positive compared to Wantai.

### Comparative, Ct value-stratified evaluation of analytical RAT sensitivity

In order to compare our data to “non-Omicron” samples [49, 50], we stratified our respiratory samples and corresponding results according to three established Ct/Cp value categories, i.e., < 25, 25–30, and > 30, similar to our previous reports [33, 42]. Compared to “non-Omicron” samples with overall sensitivities ranging between 50 and 95%, these values dropped down to 17 to 49% for BA.4 and BA.5 samples, except for nal von Minden and Clongene (Table 5). Their sensitivities were more comparable to the “non-Omicron” samples.

**Table 5:** Comparative evaluation of the analytical sensitivity of ten SARS-CoV-2 rapid antigen tests stratified for Ct/Cp value ranges based on studies by the PEI (“non-Delta/non-Omicron”*) and the current study for respiratory samples containing Omicron-BA.4 and -BA.5

	*N*	*C*_t_ < 25 (%)	*C*_t_ 25–30 (%)	*C*_t_ > 30 (%)	Overall sensitivity (%)
*iHealth*					
Non-Delta/non-Omicron*	n.a.	n.a.	n.a.	n.a.	95.0
Omicron-BA.4	71	94.3	6.7	0.0	49.3
Omicron-BA.5	100	80.5	25.6	5.0	44.0
*nal von Minden*					
Non-Delta/non-Omicron*	n.a.	83.3	13.0	0.0	36.0
Omicron-BA.4	71	77.1	3.3	0.0	39.4
Omicron-BA.5	100	82.9	5.1	0.0	36.0
*AmonMed*					
Non-Delta/non-Omicron*	n.a.	100.0	87.0	30.0	80.0
Omicron-BA.4	71	71.4	3.3	0.0	36.6
Omicron-BA.5	100	90.2	15.4	0.0	43.0
*New Gene*					
Non-Delta/non-Omicron*	n.a.	100.0	87.0	20.0	78.0
Omicron-BA.4	71	71.4	3.3	0.0	36.6
Omicron-BA.5	100	80.5	7.7	0.0	36.0
*Clongene*					
Non-Delta/non-Omicron*	n.a.	94.4	34.8	0.0	50.0
Omicron-BA.4	71	65.7	3.3	0.0	33.8
Omicron-BA.5	100	87.8	10.3	0.0	40.0
*Siemens*					
Non-Delta/non-Omicron*	n.a.	100.0	87.0	0.0	76.0
Omicron-BA.4	71	60.0	3.3	0.0	31.0
Omicron-BA.5	100	70.7	2.6	0.0	30.0
*Hotgen*					
Non-Delta/non-Omicron*	n.a.	100.0	47.8	0.0	56.0
Omicron-BA.4	71	54.3	0.0	0.0	26.8
Omicron-BA.5	10	70.7	2.6	0.0	30.3
*Joinstar*					
Non-Delta/non-Omicron*	n.a.	100.0	60.9	0.0	64.0
Omicron-BA.4	71	57.1	3.3	0.0	29.6
Omicron-BA.5	100	73.2	5.1	0.0	32.0
*Genrui*					
Non-Delta/non-Omicron*	n.a.	94.1	56.5	0.0	58.0
Omicron-BA.4	71	42.9	0.0	0.0	21.1
Omicron-BA.5	100	53.7	0.0	0.0	22.0
*Wantai*					
Non-Delta/non-Omicron*	n.a.	100.0	25.0	0.0	50.0
Omicron-BA.4	71	34.3	0.0	0.0	16.9
Omicron-BA.5	100	43.9	0.0	0.0	18.0

*n.a.* not available

*[49, 50]

Interestingly, the ten RATs showed already marked differences in the highest viral load category with Ct/Cp values < 25: The sensitivities of BA.4/BA.5 ranged between 34 and 90% compared to “non-Omicron” samples with 83 to 100%, with nal von Minden posing the only exception. In contrast, the efficacy of Wantai and Genrui to detect BA.4 and BA.5 in the highest viral load category was only between 34 and 53% compared to “non-Omicron” samples reported with sensitivities of 94 and 100%. The differences between “non-Omicron” samples were even more obvious in the intermediate viral load category with Ct/Cp values between 25 and 30. Here, the ten RATs clustered into three different groups: in the first group, Clongene and nal von Minden reached 3 to 10% compared to “non-Omicron” samples with 13 to 35%. The second category included New Gene, Siemens, and AmonMed scoring 3 to 15% positive compared to “non-Omicron” samples reported with 87%. Hotgene, Wantai, and Genrui belonged to the third group where BA.4 and BA.5 were partly or not at all detected, while “non-Omicron” samples had apparently scored positive in 25 and 57% of cases. The low viral load category with Ct/Cp > 30 was generally not recognized; only iHealth was able to recognize BA.5 in 5% of samples.

## Discussion

The Omicron VoC subvariants BA.4 and BA.5, first detected beginning of 2022 and derivatives of BA.2, spread rapidly worldwide and became the predominant SARS-CoV-2 variants within a few weeks [58, 59]. In particular, the BA.5 subvariant has dominated the epidemic in Germany since the middle of 2022 [60].

From June 2022 onwards, “citizen PCR” or RAT testing free of cost in Germany has been available only to selected groups of individuals [61], and therefore, the importance of over-the-counter RATs for non-professionals to diagnose SARS-CoV-2 infections has further increased. In the current study, ten of the most commonly sold RATs for layman’s use were evaluated for their specificity and sensitivity as a function of the viral load in specimens. To this end, two approaches were pursued: first, 171 SARS-CoV-2 RNA PCR-positive respiratory samples (100 BA.5, 71 BA.4) and, second, cell culture-expanded clinical isolates of both subvariants were studied.

While conducting this study, we encountered several methodological shortcomings in the EU common list of COVID-19 antigen tests: The guidelines consider the selection of samples in retrospective laboratory studies (category B) [45]. However, these appear to be rather imprecise and possibly error prone: First, it is recommended that samples from certain RNA concentration or Ct value ranges should be used. However, it was not further specified which volume of a clinical sample should be applied to the assay. In established and internationally accepted protocols, 50 µl of one sample per test is usually used [50], which was also applied for the current study. Yet, this lack of "RNA copies subjected to test" specification in EU guidelines may result in substantial inter-study deviations and thus errors evaluating the clinical sensitivity. Second, the recommended ranges of the naturally occurring viral loads and corresponding Ct values (“approximately 1.1 × 10^9^ to 4.2 × 10^2^ genome copies per mL of specimen and Ct values between 17 and 36”) mentioned in the guidelines of the EU common list are also specified in a publication of the Paul Ehrlich Institute (PEI) [50]. However, in this manuscript, the data of RNA concentration and Ct values (referred to by the EC) are mentioned independently of each other (in one instance as maximum and minimum concentrations of the investigated pool samples, in the other instance in the basic panel description) and are not directly related to each other. Therefore, uncertainty remains regarding the correct conversion of a Ct value measured in the laboratory into a viral load. Here, precise information and efforts to harmonize this procedure are desirable.

The investigation of RAT specificity is often omitted in current studies and only sensitivity is included in the EU common list, which has been exclusively evaluated by the PEI [45]. Yet, specificity should be determined according to the MDCG 2021–21 Rev.1 Guidance on performance evaluation of SARS-CoV-2 in vitro diagnostic medical devices using 300 samples from uninfected individuals [62]. However, manufacturer data are also accepted as an alternative, which poses an obvious conflict of interest, and results for both sensitivity and specificity provided by manufacturers have frequently not been confirmed by independent laboratories [10, 11, 33, 42, 63–67]. The EU common list does not include RATs that exclusively allow saliva as input material [45] since this can go along with reduced sensitivity [68]. Interestingly, the instructions for use enclosed with the purchased AmonMed (Ref. No. CG01AG) recommends the use of saliva, whereas the test appearing in the EU common list with the same Ref. No., but with a different device ID, was apparently evaluated for use with nasal specimen material only [45, 69]. For the nasal test, the manufacturer's data indicates a specificity of 99.55% [45]. In our specificity study, all tests were performed according to the manufacturer's instructions; thus, the specificity was measured in saliva and, with 84%, did not meet the minimum criteria of the EU common list and the WHO. Although we were only able to examine 50 instead of the recommended 300 samples, with a 95% CI of 71.49—91.66%, this is still considerably below the required minimum of 97%. However, sensitivity testing for AmonMed did not show higher detection rates at intermediate or low viral loads (0% at Ct > 30) in our study. For the sensitivity test, no directly collected swabs were applied to the extraction buffer, but virus-containing VTM was used instead, possibly diluting potentially interfering contaminants. Nevertheless, our observation suggests that substances contained in saliva may lead to false-positive results here.

The investigation of various VoCs for their performance in RATs was central to our work. The European Commission explicitly emphasizes to pay special attention to the performance of RATs in the context of emerging SARS-CoV-2 variants. However, many studies—especially the extensive evaluations of the PEI, which are often used as reference in the EU common list—date back to times of the pandemic before the emergence of Omicron VoC and its subvariants and before the establishment of widespread immunity in the population with the occurrence of breakthrough infections or multiple vaccinations [45, 50]. Several previous studies have shown divergent performance data of RATs with emerging variants [27, 33, 70, 71].

In this study, we did not detect significant differences in individual RAT’s sensitivity to detect samples containing BA.4 and BA.5. Reasons for not detecting differences in sensitivity for these recent Omicron subvariants in contrast to our previous study using samples containing either BA.1 or Delta [33] may include the following: although studies have shown that in most assays RAT extraction buffers are not able to completely inactivate SARS-CoV-2 [72, 73], the rationale suggesting that the capacity of the extraction buffer to make the nucleocapsid protein in the samples accessible to test antibodies may have an impact on the sensitivity of RATs is not evident in the current study. Comparing the lower limit of detection of, on one hand, cell culture samples, inactivated and highly denatured using Triton X-100, with, on the other hand, clinical swab samples extracted by the individual test extraction buffer, the current study showed a good correlation of these results (data not shown), i.e., the ranking of RATs’ performance. One can speculate that the influence of pre-existing immunity, which has been discussed repeatedly [32, 43], may be a key contributing factor to changing RAT performance in the course of the pandemic. Interestingly, antibody prevalence (including anti-spike antibodies) was in the range of approximately 90% in the German general population at the time of the previous study for both the sampling period of the Delta VoC study (October 30, 2021 to January 17, 2022) as well as the “Omicron VoC sampling period” (November 26, 2021 to January 19, 2022) [74, 75]. However, the prevalence of anti-nucleocapsid antibodies as an indicator of convalescence was only about 10% in Munich at that time (data from the fifth round of blood sampling of the KoCo19 study (October 2021 to January 2022)) [76, 77]. Vaccination rates in Germany during the “Delta VoC sampling period” were 2–48% for triple-vaccinated individuals and 12–50% during the Omicron VoC period [78]. Thus, the study period overlapped with the main booster vaccination period in Germany. A dramatic increase in infections (> 100,000 reported new infections per day) [79], however, only occurred later and thus after the end of the second study. A change in immunity due to a third vaccination, if any, may have had a small impact on the difference of overall vaccination rate between these two cohorts (“Delta” vs “Omicron”). Some studies suggest that the impact of the third vaccination on the induction of mucosal immunity may limited [80, 81]. Broader immunity due to higher infection rates probably played a minor role in the previous study, assuming no frequent reinfections in exposed populations. Despite this uncertainty, we consider it more likely that altered replication characteristics of the Omicron VoC and its subvariants, compared to preceding VoCs, may be a critical factor in reducing the sensitivity of RATs since late 2021. An altered ratio of excreted RNA to nucleocapsid protein may lead to increased false-negative RAT results.

The observational study presented here clearly underlines that the RATs on the market significantly differ in their sensitivity for recent Omicron subvariants, which is due to intrinsic properties of the respective test produced. Therefore, it remains the task of the supervising and regulatory authorities to manage and supervise the large market of SARS-CoV-2 RATs by appropriate and precise measures.

Another aspect that requires attention for the interpretation of these types of retrospective laboratory studies is the frequent lack of clinical information about the patients from whom respiratory samples were obtained. During the course of an infection, the amount of antigen and RNA present on the respiratory mucosa changes and therefore the time of onset of symptoms will likely affect the test results [82]. This may affect sensitivity, particularly in samples with intermediate and low viral loads. In addition to anamnestic information, no knowledge about the anatomical sampling sites was available, which is why clinical performance based on different specimen types could not be examined in the context of sensitivity testing. However, this limitation is generally accepted. Thus, RATs should constantly be evaluated by independent laboratories for their performance when new SARS-CoV-2 VoCs or sublineages are rising.

We propose that the following aspects and insights over the course of the COVID-19 pandemic from the current and our previous studies [10, 33, 42] should be considered in the context of layman’s RAT use:

1. *Test market regulation* During the last 3 years of the pandemic, to our knowledge, only one generation of RATs has been developed. Global markets were rapidly supplied with these products, and tests were introduced and approved largely without independent evaluation of performance. As the information provided to the general public was predominantly shaped by manufacturers' claims, a misleading perception regarding the reliability of RATs was unfortunately established in the public domain [83]. This includes the misperception of a high value of a negative RAT result. The extent to which commercial interests of manufacturers lagged behind scientific accuracy, responsibility in patient care, and ethical considerations during this seemingly weakly supervised period can only be speculated, and it cannot be generalized to all vendors and tests distributed. While policy makers’ commitment to the introduction of RATs was strong, little consequences were unfortunately drawn from early scientific studies, which put RATs’ performance in doubt. From our perspective, this is the biggest shortcoming of the test strategy in the COVID-19 pandemic and should be avoided in the future.
2. *Test development* Factors other than independently determined performance characteristics have apparently been shaping the sales of RATs. Regrettably, neither societies for virology or infectious diseases nor political decision-makers have exerted sufficient pressure on manufacturers to improve RATs for SARS-CoV-2 during the course of the pandemic. Our current study demonstrates that even after more than two years of distribution, the lower limits of detection (LoD) and sensitivities can drastically differ among different RATs. This supports the notion that "poor-performing tests" could have—within the general limitations of liquid chromatography-based methodology—been markedly improved. An exception to this market (mis)regulation seems to be the iHealth RAT, which performed best in our evaluation and was also a bestseller on Amazon.com in the US, but did, unfortunately, not obtain approval for the European market. It is surprising that regulatory authorities in Europe were apparently unable to identify the best performing RATs, recommend their use, and define their performance as a reference for all other RATs seeking market access.
3. *Test performance evaluation* Instead of defining objective and quantitative RAT evaluation criteria such as LoD50/95 values, a set of seemingly arbitrary criteria for diagnostic sensitivity and also specificity were implemented by regulatory authorities [84]. Unfortunately, not even official bodies, including the Paul Ehrlich Institute, applied these criteria during their evaluation of some of the tests being sold in Germany. First, specificity was not evaluated, even though some RATs clearly fail in achieving minimal requirements in this category (see, for example, Table 1 in this study). Second, the number of samples tested to determine sensitivity were too small and their viral loads frequently not representative [18, 85]. Instead, manufacturers’ information was merely copied into official tables displayed in websites of BfArM or EC, which were then accessed by the general public under the assumption of an independent validation by these official institutions.
  In the context of PCR diagnostics, the public was moderately successful in familiarizing themselves with the interpretation of cycle threshold (Ct) values. It would have been desirable to regularly characterize and adequately communicate the RAT diagnostics accessible to laypeople using the more precise and independent parameter of the lower limit of detection. Instead, the unsubstantiated claims of extremely high RAT sensitivities (frequently > 97%) published by manufacturers, distributors, and test center operators were adhered to.
4. *Assessment of benefit and risk of a negative RAT result* The ability of the general public in Germany to adequately assess the significance and potential consequences of a negative or false-negative SARS-CoV-2 RAT remained relatively low [83]. The negative predictive value, which depends on the incidence at a given time, is a complex parameter that has been shown to be somewhat unreliable even among higher-educated population groups [83]. Therefore, we share the position of the ALM e.V. (Association of Accredited Laboratories in Medicine) that the experiences associated with the scandals involving SARS-CoV-2 antigen tests in testing centers underscore the demand to maintain the requirement of supervision by physicians (Arztvorbehalt) in our regulated healthcare system [86]. This pertains to the conscientious use of SARS-CoV-2 RATs during the pandemic, which includes test selection for RATs used in testing centers, as well as the interpretation and official monitoring of the generated results.
5. *Evolution of the virus* Based on our continuous evaluation of both point-of-care RATs and laboratory-based automated antigen tests throughout the COVID-19 pandemic, we conclude multiple factors may impact their performance. These include the emergence of new variants VoCs, the development of a complex pattern of vaccine- and infection-induced immunity, clinical manifestation and severity of the disease, or dynamics of SARS-CoV-2 RNA and nucleocapsid shedding onto the mucosa. The “antibody bridging concept” proposed by the PEI has already discussed limitations [49, 50] and has failed in its prediction of RAT performance for the Omicron variant, which emerged at the end of 2021. Several laboratory-based but particularly prospective clinical studies, such as those conducted by colleagues in Würzburg [43] and Berlin [32], demonstrate that the reliability in performance of RATs is apparently multifactorial and difficult to predict.
6. *Automated SARS-CoV-2 antigen tests* In Germany, due to the early and strong promotion of PCR diagnostics alongside the introduction of RATs, there was no significant gap in demand between these two diagnostic tools that could have been filled by automated antigen tests. Although these tests are carried out on platforms in specialized laboratories where performance data can be properly assessed, it is surprising that also here no advancement in performance was observed during the pandemic, and noticeable test differences in sensitivity persisted until the end.

In summary, from a public health perspective, surveillance for SARS-CoV-2 infections using RATs is nowadays of limited utility and should not be recommended as a means to determine an individual’s infectious status. Lessons learned from the use of antigen-based point-of-care tests during the COVID-19 pandemic should be applied to future pandemics.

## Supplementary Information

Below is the link to the electronic supplementary material.

Supplementary file1 (DOCX 48 kb)

## Data Availability

Not applicable.
