# Supplementary material for: Ten rapid antigen tests for SARS-CoV-2 widely differ in their ability to detect Omicron-BA.4 and -BA.5
Source: Med Microbiol Immunol. 2023 Aug 10;212(5):323–37. doi: 10.1007/s00430-023-00775-8 (PMC10501931; doi:10.1007/s00430-023-00775-8)
Supplement: Supplementary file 1 — Supplementary file1 (DOCX 48 kb) [file 430_2023_775_MOESM1_ESM.docx]

**Suppl. Table 1:** Comparison table for converting recommended Ct value ranges to corresponding viral loads. Accordingly, viral loads are expressed in Geq/ml. The conversion formulas for the test system of the European Commission (EC) were derived from the data of the EU Common list of COVID-19 antigen tests [45] and those of the PEI from a corresponding publication [49, 50].

| Subgroup | Ct- Range {1} | | | Viral load (EC) | | | Viral load (MvPI) | | | Viral load (PEI) | | |
| --- | --- | --- | --- | --- | --- | --- | --- | --- | --- | --- | --- | --- |
| high (very high) | 17 | - | 25 | 1,10 x 10^9^ | - | 2,10 x 10^6^ | 3,50 x 10^8^ | - | 1,20 x 10^6^ | 3,24 x 10^8^ | - | 1,33 x 10^6^ |
| medium (high) | 25 | - | 30 | 2,10 x 10^6^ | - | 4,40 x 10^4^ | 1,20 x 10^6^ | - | 3,30 x 10^4^ | 1,33 x 10^6^ | - | 4,27 x 10^4^ |
| low (medium) | 30 | - | 36 | 4,40 x 10^4^ | - | 4,10 x 10^2^ | 3,30 x 10^4^ | - | 4,60 x 10^2^ | 4,27 x 10^4^ | - | 6,91 x 10^2^ |
| conversion formula |  |  |  | f(Ct) = e^(43,738-Ct)/1,285^ | | | f(Ct) = e^(44,576-Ct)/1,401^ | | | f(Ct) = e^(41,154-Ct)/1,455^ *20 {2} | | |

{1} as defined by EC and PEI

{2} The factor 20 must be added because the PEI data refer to RNA copies contained in a 50µl aliquot subjected to one test

MvPI: Max von Pettenkofer-Institute

PEI: Paul-Ehrlich-Institute

**Suppl. Table 2:** Summary of non-synonymous amino acid substitutions in the SARS-CoV-2 nucleocapsid protein with >75% prevalence in publicly available VoC sequences compared to the original Wuhan-hu-1 sequence. [87]

| Amino acid position | D3 | P13 | DEL30/32 | DEL31/33 | S33 | D63 | P80 | E136 | P151 | R203 | G204 | T205 | G215 | S235 | D377 | S413 |
| --- | --- | --- | --- | --- | --- | --- | --- | --- | --- | --- | --- | --- | --- | --- | --- | --- |
| Alpha (B.1.1.7) | L |  |  |  |  |  |  |  |  | K | R |  |  | F |  |  |
| Beta (B.1.351) |  |  |  |  |  |  |  |  |  |  |  | I |  |  |  |  |
| Gamma (P.1) |  |  |  |  |  |  | R |  |  | K | R |  |  |  |  |  |
| Delta (B.1.617.2) |  |  |  |  |  | G |  |  |  | M |  |  | C |  | Y |  |
| Omicron (BA.1.1) |  | L |  | del* |  |  |  |  |  | K | R |  |  |  |  |  |
| Omicron (BA.2) |  | L |  | del* |  |  |  |  |  | K | R |  |  |  |  | R |
| Omicron (BA.2.75) |  | L |  | del* |  |  |  |  |  | K | R |  |  |  |  | R |
| Omicron (BN.1) |  | L |  | del* |  |  |  |  |  | K | R |  |  |  |  | R |
| Omicron (XBB.1) |  | L |  | del* |  |  |  |  |  | K | R |  |  |  |  | R |
| Omicron (BA.4) |  | L |  | del* |  |  |  |  | S | K | R |  |  |  |  | R |
| Omicron (BA.5) |  | L |  | del* |  |  |  |  |  | K | R |  |  |  |  | R |
| Omicron (BQ.1) |  | L |  | del* |  |  |  | D |  | K | R |  |  |  |  | R |
| Omicron (BF.7) |  | L | del* |  | F |  |  |  |  | K | R |  |  |  |  | R |

*deletion

**Suppl. Table 3** Detailed information of the ten SARS-CoV-2 RATs examined in this study.

| Study Alias | New Gene | iHealth | Clongene | nal von minden | Hotgen | Joinstar | Siemens | AmonMed | Wantai | Genrui |
| --- | --- | --- | --- | --- | --- | --- | --- | --- | --- | --- |
| Manufacturer | New Gene (Hangzhou) Bioengineering Co., Ltd. | iHealth Labs Inc. | Hangzhou Clongene Biotech Co., Ltd. | nal von minden GmbH | Beijing Hotgen Biotech Co., Ltd. | Joinstar Biomedical Technology Co., Ltd | Healgen Scientific Limited Liability Company (Siemens Healthineers) | Xiamen AmonMed Biotechnology Co., Ltd. | Beijing Wantai Biological Pharmacy Enterprise Co., Ltd. | Genrui Biotech Inc. |
| Test name | Covid-19-Antigen-Testkit | iHealth COVID-19 Antigen Rapid Test | COVID-19 Antigen Rapid Test | NADAL COVID-19 Ag Test | Novel Coronavirus 2019-nCoV Antigen Test (Colloidal gold) | COVID-19 Antigen Schnelltest (Colloidal Gold) | CLINITEST® Rapid COVID-19 Antigen Test | COVID-19 Antigen Rapid Test Kit (Colloidal Gold) | Wantai SARS-CoV-2 Ag Schnelltest (Kolloidales Gold) | Genrui SARS-CoV-2 Antigen Test Kit (Colloidal Gold) |
| Ref. No. | COVID-19-NG21 | - | ICOV5002-B025 | 243103 | HGCG134S0101 | RPBH19420 | GCCOV-502a | CG01Ag-01S-ST | WJ-2901 | 52104097 |
| Ref. No. ECL | COVID-19-NG08 | - | ICOV5002-B025 | 243103 | AT120/20 (Model A/B) | - | GCCOV-502a | CG01Ag-25 | WJ-2950 | 52025-52027(…), 52104(…),52112(…),52129(…) |
| AT-No. | AT331/21 | EUA210470 | AT079/20 | AT021/20 | AT120/20 | AT236/20 | AT001/20 | AT246/21 | AT1265/2 | AT111/20 |
| AT-No. Self-test | AT1210/21 |  |  | AT1293/21 | AT1236/21 | AT1276/21 | AT1208/21 | AT1279/21 | AT1265/21 | AT1200/21 |
| Device identification (European Commission) | 2303 | - | 1363 | 2848 | 2807 | 1333 | 1218 | 1763 | 1485 | 2012 |
| Device ID 04.12.2022 | 1501 | - | 1363 | 1162 | 1870 | 1333 | 1218 | 1763 | 1485 | 2012 |
| ECL category at time of study | B.1 | - | B.1 | - | B.1 | B.1 | A.1 | B.1 | A.1 | B.1 |
| ECL category 04.12.2022 | A.1 | - | B.1 | A.1 | B.1 | B.1 | A.1 | B.1 | A.1 | B.1 |
| Used antibodies | monoclonal | monoclonal | monoclonal | monoclonal | monoclonal | monoclonal | monoclonal | monoclonal | monoclonal | monoclonal |
| Detected antigen | nucleocapsid protein | nucleocapsid protein | nucleocapsid protein | nucleocapsid protein | nucleocapsid protein | nucleocapsid antigen | nucleocapsid, spike protein | nucleocapsid protein | nucleocapsid protein | nucleocapsid protein |
| Recommended material | Anterior nasal swab, Nasal swab | anterior nasal swab | Nasal swab, nasopharyngeal swab, oropharygeal swab | anterior nasal swab | Anterior nasal swab | Nasal swab, Nasopharyngeal swab, Oropharyngeal swab | Nasal swab, Nasopharyngeal swab | Saliva | Nasal swab, Nasopharyngeal swab | Nasal swab, Nasopharyngeal swab, Oropharyngeal swab |
| PEI evaluated | yes | no | yes | yes | yes | yes | yes | yes | yes | yes |
| PEI Bridging | yes | - | yes | yes | yes | yes | yes | yes | yes | yes |
| Intended by the manufacturer for self-testing ("self-tests") | yes | yes | no (for healthcare professionals) | no (only for professional use) | yes | yes | no (for medical professional use only) | yes | yes | yes |
| Manufacturer sensitivity | 96.8 % (Antigen) | 94,3% (positive percent agreement) | 95.5% (nasal swab) | 94.1 % (Ct<30) | 96.95 % (Antigen) | 96.1 % | 97.25 % (Nasal swab) | 98.02% | 93.22 % | 91.15 % (Nasal Swab) |
| Manufacturer specificity | 99.1 % (Antigen) | 98,1% (negative percent agreement) | 100% (nasal swab) | 99.9 % (>99.9 %) | 98.88 % (Antigen) | 98.1 % | 100 % (Nasal swab) | 99.55% | 98.96 % | 99.02 % (Nasal Swab) |
| Manufacturer Limit of detection (TCID50) |  | 20*10^3 TCID50/mL | 5,7*10^2 TCID50/ml |  | TCID50/ml Limit of detection is 5*10^1.2TCID50/mL |  | 1,15*10^2 TCID50/mL | 500 TCID50/ml | 137 TCID50/ml |  |
| Limit of detection (Protein Concentration) | 0.05 ng/mL |  |  | 0.4 ng/mL |  | 100 pg/mL |  |  | 25 pg/mL | 150 AU |
| Storage before testing | use directly | up to 1h | up to 1h | use directly | use directly | use directly | up to 1h | up to 1h | up to 30min | up to 1h |
| Extraction protocol | insert swab tip 2-3cm into one nasal cavity, rotate 5 rounds for 20s, repeat on other side, put swap into extreaction tube, stir for 5 rounds, squeeze swap with fingers, move swap up and down 3 times, insert tube cap and squeeze the tube 3 times to mix the solution, put the tube still for 1min | insert swab in nose (1/2 to 3/4 inch), brush 5 times against the nasal wall for 15s, insert swab into tube, stir 15 times, squeeze when removing the swab | insert swab 2,5 cm into nostril, rotate 5times, repeat on other side, insert swab in extraction reagent and roll 5 times, leave the swab for 1min, remove the swab while squeezing the tube, | insert swab into nostril, rotate 5 times, withdraw while continuing to rotate, repeat on other side, insert buffer in tube, swirl swab 10-15 times by compressing the wall | insert swab 1,5cm into nostril, rotate5-6 times for 15s, repeat on other side, soak the swab in buffer for 15s | insert the swab 2-2,5cm into nostril, rotate 5 times, repeat on other side, rotate swab 10 times in extraction buffer, squeeze the wall whilst removing the swab, | insert swab 2-4 cm, roll swab 5 times along mucosa, repeat on other side, add 10 drops (0,3ml) of extraction buffer into extraction tube, insert swab and roll 6times, leave the swab for 1min, squeeze whilst removing the swab | insert swab in into mouth cavity, place saliva swab between upper and lower molar teeth, gently bite the swab for 10s, stir the saliva swab in the extraction tube for more than 5 times, leave the swab in the tube for 1min | insert entire swab into nostril, rotate 4 times for 15s, repeat on other side, place swab in extraction buffer an rotate, break swab at break line | insert swab 2cm into nostril, rotate 5 times for 15s, put the swab in the extraction puffer and roll 5-6 times, squeeze whilst removing, leave the swab in the extraxction puffer for 1min, seal the extraction tube an shake 10 times |
| Drops per test | 3 drops | 3 drops | 3 drops | 2 drops | 4 drops | 2 drops | 4 drops | 2 drops | 3 drops | 3-4 drops (0,1mL) |
| Incubation time | 15-30min | 15-30min | 15-20min | 15min | 15-30min | 15-20min | 15-20min | 15-20min | 15-30min | 15-20 min |
| package leaflet | [COVID-19 Antigen Detection Kit - Nasal Swab for 1Test \| NEWGENE (new-gene.net)](https://www.new-gene.net/archives/1333) | <https://ihealthlabs.com/pages/ihealth-covid-19-antigen-rapid-test-details> | [Clungene-Laientest-Anleitung.pdf (terencebud.de)](https://www.terencebud.de/media/pdf/11/01/35/Clungene-Laientest-Anleitung.pdf) | <https://www.nal-vonminden.com/pdf/Gebrauchsanweisungen%20NADAL%20COVID-19%20Ag%20Test%20243117%20_CE0197.pdf> | <http://www.hotgen.com.cn/Uploads/Picture/2021/07/09/u60e84586878d2.pdf> | [73d0ca_4d8b504c57384f70a57d6b4231089091.pdf (joinstar-iedau.com)](https://www.joinstar-iedau.com/_files/ugd/73d0ca_4d8b504c57384f70a57d6b4231089091.pdf) | [Rapid-COVID-19-Antigen-Test-PI-Healgen-CE-20210226_B21986-09_Rev.-G_Final--1-.pdf (siemens-healthineers.com)](https://marketing.webassets.siemens-healthineers.com/b125e51689f8cda5/c8017fdbf518/Rapid-COVID-19-Antigen-Test-PI-Healgen-CE-20210226_B21986-09_Rev.-G_Final--1-.pdf) | <http://en.amonmed.com/upload/file/202205/20220507143549_89076.pdf> | [新冠抗原彩页 (ystwt.cn)](https://www.ystwt.cn/wp-content/uploads/2020/11/Wantai-SARS-CoV-2-Antigen-Rapid-Test-Brochure.pdf) | <https://manuals.plus/genrui/sars-cov-2-antigen-test-kit-manual-2.pdf> |
